# The prognostic value of radiogenomics using CT in patients with lung cancer: a systematic review

**DOI:** 10.1186/s13244-024-01831-4

**Published:** 2024-10-28

**Authors:** Yixiao Jiang, Chuan Gao, Yilin Shao, Xinjing Lou, Meiqi Hua, Jiangnan Lin, Linyu Wu, Chen Gao

**Affiliations:** 1 Department of Radiology, The First Affiliated Hospital of Zhejiang Chinese Medical University (Zhejiang Provincial Hospital of Chinese Medicine), Hangzhou, China; 2 The First School of Clinical Medicine, Zhejiang Chinese Medical University, Hangzhou, China

**Keywords:** Lung cancer, Radiomics, Genomics, Prognosis

## Abstract

**Abstract:**

This systematic review aimed to evaluate the effectiveness of combining radiomic and genomic models in predicting the long-term prognosis of patients with lung cancer and to contribute to the further exploration of radiomics. This study retrieved comprehensive literature from multiple databases, including radiomics and genomics, to study the prognosis of lung cancer. The model construction consisted of the radiomic and genomic methods. A comprehensive bias assessment was conducted, including risk assessment and model performance indicators. Ten studies between 2016 and 2023 were analyzed. Studies were mostly retrospective. Patient cohorts varied in size and characteristics, with the number of patients ranging from 79 to 315. The construction of the model involves various radiomic and genotic datasets, and most models show promising prediction performance with the area under the receiver operating characteristic curve (AUC) values ranging from 0.64 to 0.94 and the concordance index (C-index) values from 0.28 to 0.80. The combination model typically outperforms the single method model, indicating higher prediction accuracy and the highest AUC was 0.99. Combining radiomics and genomics in the prognostic model of lung cancer may improve the predictive performance. However, further research on standardized data and larger cohorts is needed to validate and integrate these findings into clinical practice.

**Critical relevance statement:**

The combination of radiomics and genomics in the prognostic model of lung cancer improved prediction accuracy in most included studies.

**Key Points:**

- The combination of radiomics and genomics can improve model performance in most studies.
- The results of establishing prognosis models by different methods are discussed.
- The combination of radiomics and genomics may be helpful to provide better treatment for patients.

**Graphical Abstract:**

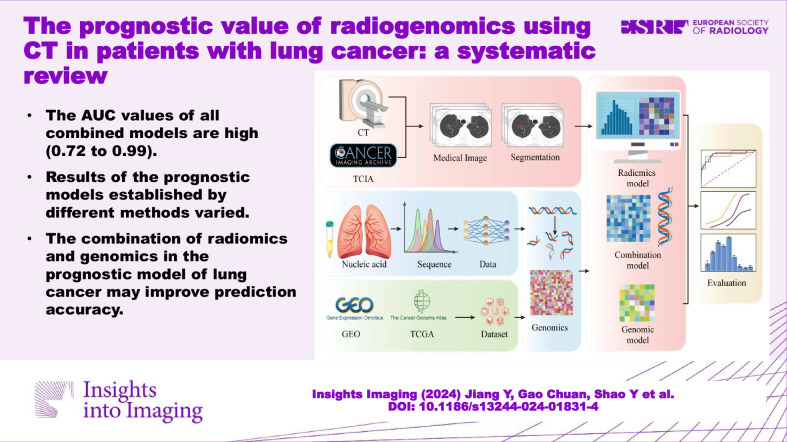

## Introduction

Lung cancer is one of the most prevalent cancers and the most common cause of cancer-related mortality worldwide [1, 2]. The overall survival (OS) rate for lung cancer has shown an increase, but it remains at only 25% [3, 4]. Therefore, accurate prognostic evaluation for cancer has become necessary in clinical practice [5, 6].

Radiomics can extract many image features from radiographic images with high throughput and then apply features to develop models and predict the phenotype of lesions non-invasively [7, 8]. Many studies have confirmed that radiomic features can be applied to the prognosis of lung cancer, such as predicting biomarkers and decoding tumor phenotypes [9–11]. Although radiomics has great clinical potential, the performance of most radiomic models still faces challenges [12, 13].

Radiogenomics assesses multi-scale links between medical imaging, genomic and clinical data [14]. It combines high-dimensional data of genomics and radiomics of tumors to develop diagnosis, prognosis, or treatment response models [15, 16]. Radiogenomics can not only aid in treatment selection for patients with lung cancer, but also provide accurate prognostic information for patients with lung cancer [17–20]. For example, Zhou et al integrated semantic image features at computed tomography (CT) with next-generation ribonucleic acid (RNA) sequencing data to create a radiogenomic map and identified radiogenomic biomarkers of non-small cell lung cancer [21]. However, as radiogenomics is an emerging field, a standard measure of research has not been conducted. Therefore, the evaluation of models’ performance became of great interest [22].

**Fig. 1:**
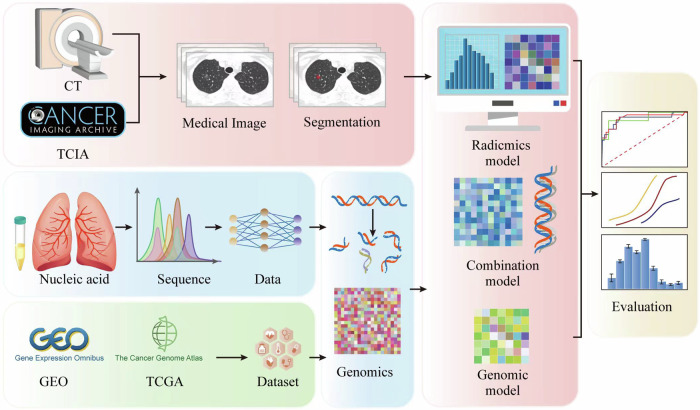
A flowchart of this study

In this review, we aimed to investigate whether the models constructed by the combination of radiomics and genomics are more effective in predicting the long-term prognosis of patients with lung cancer than models relying on either method alone, which may strengthen the relationship between radiogenomics and clinical practice. Figure 1 presents a flowchart of this study.

## Materials and methods

This systematic review was reported according to the Preferred Reporting Items for systematic reviews and meta-analysis guidelines [23]. The review was registered on PROSPERO before initiation (registration no. <CRD42023472571>).

### Search strategy

The PubMed, Embase, Web of Science Core Collection, and Cochrane Library databases were comprehensively searched up to May 13, 2024, to identify studies that used radiomics and genomics to evaluate the prognosis in patients with lung cancer. The reference lists of the included articles and the relevant literature were also manually searched. The following basic search terms were used: lung cancer, radiomics, genomics, transcriptomics, radiogenomics, and prognosis. Only original articles were considered for analysis, and there was no limit on the year or language of publication.

### Study selection

Original research articles were included in the study. Eligibility criteria included the following: (1) lung cancer patients who underwent CT imaging; (2) the construction of a model that combines genomics and radiomics to predict prognosis; and (3) including the detailed combination method. Studies were excluded if they (1) were case studies, editorials, letters, review articles, and conference abstracts; (2) were not in the field of interest; or (3) articles with missing data or overlapping patients.

### Data extraction

Data to be extracted included the following: (1) study details: first author, publication year, country, study design, cohort, sample size, histological subtype, staging, prognostic outcome, and follow-up time; (2) CT image and radiomics details: slice thickness, segmentation methods, feature extraction details, and radiomic model details; (3) genomics details: data source, methods of gene feature selection, and genomics model details; and (4) combination details: the data used to build the model, methods of combination, and the corresponding performance. The details of the acquisition parameters of the images, features details, and a number of genomic features are included in Supplementary Tables 3 and 4.

### Risk of bias assessment

Bias risk assessment assessed the methodological quality of each study using a radiomics quality score (RQS) and the predictive model bias risk assessment tool (PROBAST) [24, 25]. Two evaluators (Y.J. and Chuan G.) independently evaluated the risk of bias and discussed differences with the third author (Chen G.). RQS consists of 16 standards, each of which can achieve a certain number of scores. PROBAST evaluates 20 questions and applicability in four areas: participants, predictors, outcome, and analysis, and each question was answered with “yes/probably yes,” “no/probably no,” or “no information.” The standard for the number of participants is that each model element should have at least ten events marked as YES. In the overall evaluation, the area is considered “low-risk” only if all questions were answered “yes” or “maybe.” The overall verdict is unclear when a question is judged as unclear, and other questions are low-risk.

## Results

### Literature search and data extraction

**Fig. 2:**
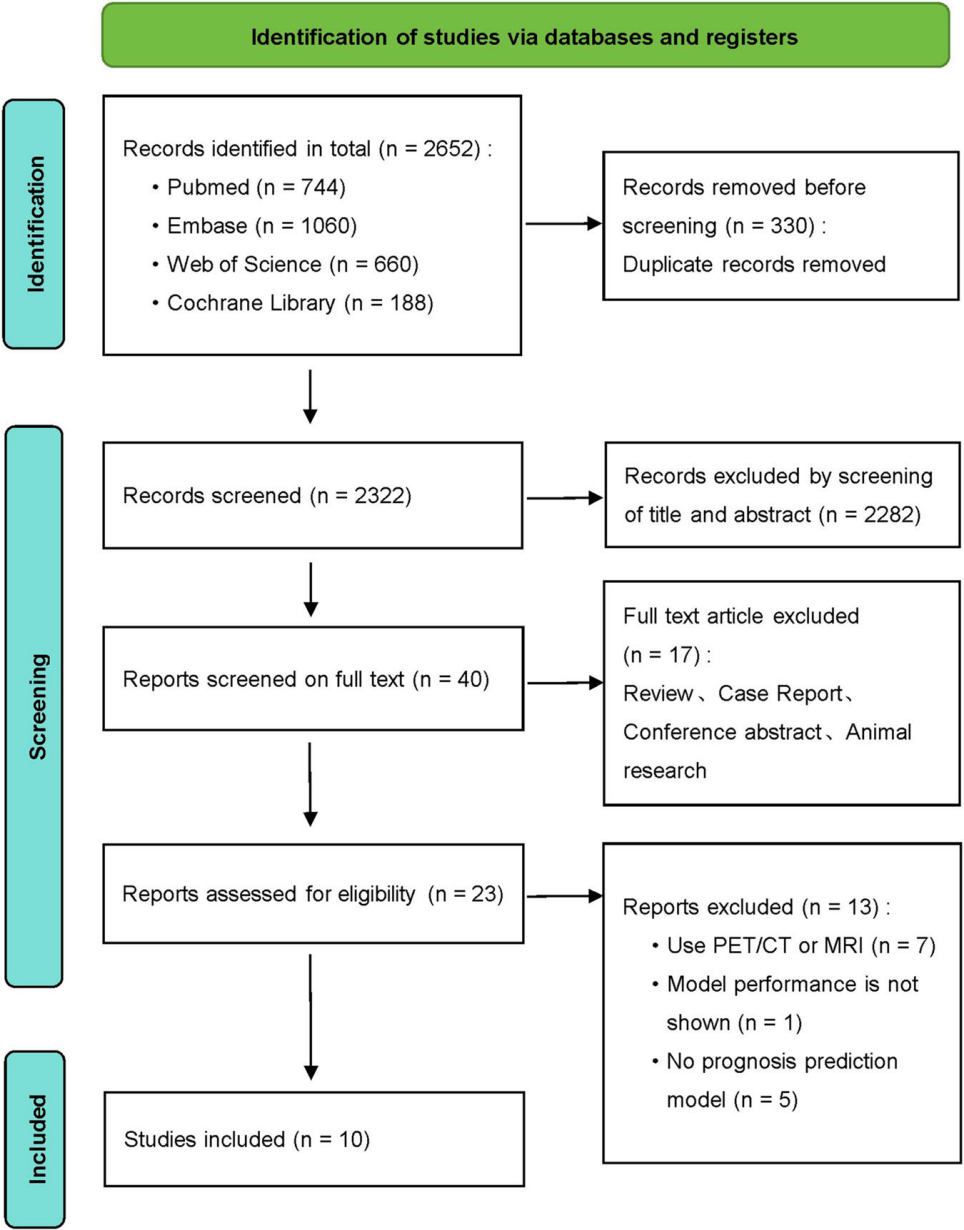
The process of our search strategy

The flow diagram of the systematic review is shown in Fig. 2. After applying the inclusion and exclusion criteria, a total of ten initially retrieved articles were included in the study.

### Patient and study characteristics

The ten included articles were published from 2016 to 2023 [26–35]. The patient characteristics of the ten studies are summarized in Table 1. Most studies (9/10, 90%) [26–28, 30–35] were retrospectively designed, except one which was prospective [29]. All articles had multiple cohorts, such as training, testing, and validation groups. There were various prognostic outcomes, including disease-free survival (DFS), objective response rate (ORR), OS, progression-free survival (PFS), recurrence, and three studies contained more than one outcome [26, 29, 35]. Genetic data from three articles (3/10, 30%) were available on Gene Expression Omnibus [27, 31, 33]. In addition, one immunotherapy-related article contained patients receiving treatment according to their treatment plans [33].

**Table 1:** Basic characteristics of patients

First author	Year of publication	Country	Study design	Cohort			Sample size			Histological subtype	Staging	Prognostic outcome	Follow-up time, (months)
				Training	Validation	External	radiomics	genomics	combination				
Nastaran Emaminejad [26]	2016	USA	R	79		/	79			NSCLC	I	Recurrence, DFS	36
Patrick Grossmann [27]	2017	USA	R	262^a^	89^b^	/	351^c^			NSCLC	I–IV	OS	32, 31^d^
Vaishnavi Subramanian [28]	2020	USA	R	107		/	107			NSCLC	/	Recurrence	/
Liyuan Fan [29]	2020	China	P	103	71	/	174			NSCLC	I–IV	ORR, PFS, and OS	40
Ki Hwan Kim [30]	2020	South Korea	R	124		/	124			NSCLC	I–IV	DFS	36 (18, 175)^e^
Wei Chen [31]	2022	China	R	87	29	/	116			NSCLC	/	OS	/
Apurva Singh [32]	2022	USA	R	3/4^f^	1/4^f^	/	85			NSCLC	I–III^g^	OS	/
Amine Bouhamama [33]	2023	France	R	110	85	/	195	54		NSCLC	II–IV	PFS	63 days^h^
Qi‐Kun Guo [34]	2023	China	R	129	51	/	180	28	180	LUAD	I–III	OS	≥ 60
Eleftherios Trivizakis [35]	2023	Greece	R	115		/	115			NSCLC	/	OS and adjuvant chemotherapy response	/

*R* retrospective study, *P* prospective study, *DFS* disease-free survival (the time from the surgical resection date until the date of relapse, which refers to tumor recurrence), *LUAD* lung adenocarcinoma, *NSCLC* non-small cell lung cancer, *ORR* objective response rate (the radiotherapy response of the primary sites was evaluated according to RECIST 1.112), *OS* overall survival, *PFS* progression-free survival (the time between surgical resection and tumor progression or death (for any reason))

^a^ Dataset1

^b^ Dataset2

^c^ Dataset1 + Dataset2

^d^ Median: 32 months (Dataset1), 31 months (Dataset2)

^e^ 18 months (mini), 36 months (median), 175 months (max)

^f^ Approach 3: 4/3 of the cases for training and 1/4 for testing

^g^ I/II/III/unspecified

^h^ Median: 63 days (PFS)

### The method of constructing models

The study details of the radiomics workflow, including acquisition parameters of the images, the region of interest (ROI) segmentation, feature extraction, selection, and a number of features are summarized in Table 2. One study [32] did not mention the slice thickness, and the other nine studies’ slice thickness ranged from 0.625 to 5 mm. The ROI segmentation method was not mentioned in one article [29], manual in two studies [27, 33], semiautomatic in five studies [26, 27, 30, 32, 34], and the remaining three were uncertain [28, 31, 35]. The number of extracted radiomic features ranged from 35 to 2996. The number of radiomic features in the final radiomic model ranged from 2 to 210. A total of eight articles constructed an independent radiomic model [26–28, 30, 31, 33–35]. The model constructed in the final radiomic model was usually a Cox proportional hazard model. More information is shown in Table 3.

**Table 2:** The details of the radiomics workflow

First author	Slice thickness, (mm)	Segmentation method	Number of extraction features	Dimension reduction method	Number of features obtained
Nastaran Emaminejad [26]	2	Semiautomatic	35	A CFS subset evaluator with a best-first heuristic feature search and selection method	8
Patrick Grossmann [27]	1.5–5.0^a^	Manual^b^ and semiautomatic^a^	636	Bi-clustering	210
Vaishnavi Subramanian [28]	0.625–3.0^c^	/^d^	/	/	/
Liyuan Fan [29]	1.5–2.5	/	52	ICC and LASSO method	22
Ki Hwan Kim [30]	1–5^e^	Semiautomatic	161	LASSO method	2/12^f^
Wei Chen [31]	0.625–3.0^c^	/^d^	/	LASSO method	8
Apurva Singh [32]	/	Semiautomatic	102	Approach 1: intra-modal feature selection and inter-modal feature selection^g^, Approach 2: consensus clustering, the first PC, Approach 3: LASSO method^h^	42/19^i^, 8^j^, 3^k^
Amine Bouhamama [33]	1–3	Manually	342	Approach 1: the ReliefF algorithm, Approach 2: statistical method accounting for relevancy and redundancy	30
Qi‐Kun Guo [34]	1	Semiautomatic	1409	ICC, LASSO method	7
Eleftherios Trivizakis [35]	0.625–3.0^c^	**/**^d^	2996	A zero-variance threshold, ANOVA, LASSO method	12/13^l^

*ANOVA* analysis of variance, *CFS* correlation feature selection, *ICC* intraclass correlation coefficient, *LASSO* least absolute shrinkage and selection operator, *PC* principal component

^a^ Dataset2

^b^ Dataset1

^c^ 1.5 (median)

^d^ The part of the original dataset that belongs to uses automatic segmentation

^e^ 2.33 (mean)

^f^ 2 (pre-contrast CT scan)/12 (post-contrast CT scan)

^g^ The Spearman correlation coefficient < 95% (intra-modal feature), non-skewed distribution, positive mean decrease in accuracy. The Spearman correlation coefficient < 95% (inter-modal feature selection)

^h^ Find optimal λ for LASSO regularization. Use λ to get the optional feature subset from the penalized Cox model

^i^ Approach 1: (intra-modal feature) 42, (inter-modal feature) an optimal multi-modal feature group (19 radiomic and 11 genomic features)

^j^ Approach 2: an optimal multi-modal feature group (eight radiomic and seven genomic representative features)

^k^ Approach 3: an optimal multi-modal feature group (three radiomic and one genomic feature)

^l^ Six radiomic features and six deep features for therapy response, six radiomic features and seven deep features for OS

**Table 3:** The details of the radiomic models

First author	Radiomic classifiers/models	Method of constructing radiomic model	The corresponding performance of the radiomic model	Cut off	Classified group
Nastaran Emaminejad [26]	Model	A standard leave-one-case-out validation method	AUC = 0.78 with a 95% CI of [0.66, 0.88]	Middle score	High/low-risk groups
Patrick Grossmann [27]	Model	A Cox proportional-hazards model	(Dataset2) *C*-index = 0.60	/	Cluster 1–13
Vaishnavi Subramanian [28]	Model	Linear models: Cox proportional hazard model with regularization; Non-linear model: train single modality MLPs	*C*-index: 0.46, 0.39, 0.30, 0.37, 0.80^a^ *C*-index: 0.55, 0.43, 0.28, 0.68, 0.47^b^	/	/
Liyuan Fan [29]	/	/	/	/	/
Ki Hwan Kim [30]	Model (clinical + radiomic data)	Multivariate Cox proportional hazards analysis, leave-one-out cross-validation	AUC = 0.8355^c^, AUC = 0.8599^d^	/	/
Wei Chen [31]	Model	LASSO Cox models	*C*-index: 0.79^e^, 0.643^f^; Cross-validation *C*-index: 0.65 ± 0.028	The median value of the risk score (training cohort)	High/low-risk groups
Apurva Singh [32]	/	/	/	/	/
Amine Bouhamama [33]	Model	SVM; An artificial neural network	AUC: 0.94^e^, 0.65^g^	/	/
Qi‐Kun Guo [34]	Model	LASSO-Cox method	/	/	/
Eleftherios Trivizakis [35]	Model	Cox, Cox proportional hazards, extra trees, survival tree, random forest, and SVM-based	AUC: 0.68 ± 0.1^h^, 0.71 ± 0.08^i^, 0.69 ± 0.1^j^, 0.69 ± 0.09^k^ *C*-index: 0.63 ± 0.08^l^, 0.68 ± 0.03^m^, 0.73 ± 0.07^n^, 0.76 ± 0.06^o^	/	/

*AUC* area under the receiver operating characteristic curve, *CI* confidence interval, *C*-index concordance index, *LASSO* the least absolute shrinkage and selection operator, *MLPs* multi-layer perceptrons, *SVM* support vector machine

^a^ Linear model (Cox)

^b^ Non-linear model (multi-layer perceptron)

^c^ Pre-contrast CT

^d^ Post-contrast CT

^e^ Training

^f^ Testing

^g^ Validation

^h^ Radiomics

^i^ Radiomic score

^j^ Deep features

^k^ Deep feature score

^l^ The best performance of radiomics (extra trees)

^m^ The best performance of radiomic score (SVM-based)

^n^ The best performance of deep features (SVM-based)

^o^ The best performance of deep feature score (SVM-based)

Table 4 details the gene analyses. Nearly every article applied unique methods to selecting genotic features. Five articles constructed an independent genomic model [26, 28, 30, 31, 35]. However, the genomic approach used in one of the articles may also elucidate certain biological biases [33].

**Table 4:** The details about the genomic models

First author	Gene source	Selection method	Genomic models/classifiers	Method of constructing genomic model	The corresponding performance of the genomic model
Nastaran Emaminejad [26]	IHC of surgical resection specimen of 79 patients (ERCC1 and RRM1)	/	Model	A Naïve Bayesian network-based classifier	AUC = 0.68 with 95% CI of [0.56, 0.79]
Patrick Grossmann [27]	Gene expression data from single-needle biopsies of Dataset1 (*n* = 262) and surgically resected tissue of Dataset2 (*n* = 89)	/	/	/	/
Vaishnavi Subramanian [28]	Gene expression data from surgical resection tumor samples (NSCLC radiogenomics dataset)	Selected the top 500 variant gene	Model	Cox proportional hazard model with regularization; Train single modality MLPs	*C*-index: 0.52, 0.45, 0.60, 0.72, 0.75^a^; *C*-index: 0.52, 0.43, 0.64, 0.50, 0.64^b^
Liyuan Fan [29]	miRNA from radioresistant cell lines (A549-R and PC9-R)	Differentially expressed miRNA profile, Fold changes, *q*-value, and relative expression value, Top-ranked	/	/	/
Ki Hwan Kim [30]	DNA from surgical resection tissues	A custom-designed filtering step, LASSO method	Model (clinical + genomic data)	Multivariate Cox proportional hazards analysis, Leave-one-out cross-validation	AUC = 0.8497^c^, 0.8376^d^
Wei Chen [31]	Gene expression data from surgical resection tumor samples (NSCLC radiogenomics dataset)	Autoencoder	Model	/	*C*-index: 0.716^e^, 0.581^f^ Cross-validation *C*-index: 0.606 ± 0.081
Apurva Singh [32]	Gene expression data from surgical resection tumor samples (NSCLC–radiomics–genomics dataset)	Selected genes that represent the three major co-expression patterns	/	/	/
Amine Bouhamama [33]	Targeted RNA sequencing was performed on patients with at least one FFPE sample from a pre-treatment biopsy who received nivolumab or pembrolizumab treatment using HTG technology (GSE161537)	/	/	/	/
Qi‐Kun Guo [34]	Sixty pairs of fresh tumors and adjacent normal tissues	/	/	/	/
Eleftherios Trivizakis [35]	Gene expression data from surgical resection tumor samples (NSCLC radiogenomics dataset)	ANOVA, LASSO method	Model	Cox, Cox proportional hazards, extra trees, survival tree, random forest, and SVM-based	AUC: 0.64 ± 0.11^g^, 0.66 ± 0.1 ^h^; *C*-index: 0.71 ± 0.15^i^, 0.72 ± 0.09^j^

*ANOVA* analysis of variance, *AUC* area under the receiver operating characteristic curve, *CI* confidence interval, *C*-index concordance index, *DNA* deoxyribonucleic acid, *FFPE* formalin-fixed paraffin-embedded, *IHC* immuno-histochemistry, *LASSO* the least absolute shrinkage and selection operator, *MLPs* multilayer perceptrons, *NSCLC* non-small cell lung cancer, *RNA* ribonucleic acid, *SVM* support vector machine

^a^ Linear model (Cox)

^b^ Non-linear model (multi-layer perceptron)

^c^ Pre-contrast CT

^d^ Post-contrast CT

^e^ Training

^f^ Testing

^g^ Transcriptomics

^h^ Transcriptomic score

^i^ The best performance of transcriptomics (SVM-based)

^j^ The best performance of transcriptomic score (SVM-based)

### The performance of the models

The details of the combination models and the corresponding performance metrics in the included studies were summarized in Table 5.

**Table 5:** The workflow of the combination models

First author	Combination data	Method of combination	The corresponding performance of combination model
Nastaran Emaminejad [26]	Quantitative image model + genomic model	Combined the classifiers’ prediction risk scores	AUC = 0.84 ± 0.05
Patrick Grossmann [27]	Clinical model + genotic model + radiomic model	Cox proportional-hazards regression model	*C*-index = 0.73
Vaishnavi Subramanian [28]	Genomic features + radiomic features + deep learning features; Genomic model + radiomic model + deep learning model	Linear models: Cox proportional and hazards model; Non-linear model: MLPs	*C*-index: 0.81^a^, 0.81^b^, 0.72^c^, 0.83^d^, 0.63^e^, 0.50^f^
Liyuan Fan [29]	Radiomic features + genomic features	Multivariable logistic regression analysis for ORR, Cox proportional hazards model for OS, PFS	*C*-index (ORR): 0.86 [95% CI: 0.75–0.92]^g^, 0.81 [95% CI: 0.69–0.89]^h^; *C*-index (OS and PFS): NA
Ki Hwan Kim [30]	Radiomic features + genotic features + clinical data	Multivariate Cox proportional hazards analysis, Leave-one-out cross validation	AUC = 0.8638^i^, 0.8672^j^
Wei Chen [31]	Radiomic model + genomic model + clinical features	Multivariate Cox proportional hazard model	*C*-index: 0.85^g^, 0.736^k^; Cross-validation *C*-index: 0.749 ± 0.044
Apurva Singh [32]	Radiomic features + genomic features + clinical features	Unsupervised hierarchical clustering, Multivariate Cox proportional hazards model	Non-cross-validated *C*-index: 0.63^l^, 0.60^m^, 0.63^n^; Five-fold cross-validated *C*-index, 95% CI: 0.61 [0.55–0.63]^m^, 0.54 [0.49, 0.60]^n^, 0.59 [0.37, 0.78]^o^
Amine Bouhamama [33]	Genomic features + radiomic features	CNN, Decision trees, SVM	AUC = 0.95^o^, 0.99^p^
Qi‐Kun Guo [34]	Clinical features + radiomic model	Multivariate Cox proportional hazard model	*C*-index: 0.815 [95% CI: 0.756–0.874]
Eleftherios Trivizakis [35]	Genomic features + radiomic features + deep learning features; Genomic model + radiomic model + deep learning model	Cox, Cox proportional hazards, extra trees, survival tree, random forest, SVM-based	AUC: 0.72 ± 0.08^q^, 0.74 ± 0.06^r^; *C*-index: 0.76 ± 0.08^s^, 0.79 ± 0.03^t^

*AUC* area under the receiver operating characteristic curve, *CI* confidence interval, *C-index* concordance index, *CNN* convolutional neural network, *MLPs* modality multi-layer perceptrons, *ORR* objective response rate, *OS* overall survival, *PFS* progression-free survival, *SVM* support vector machine

^a^ The best performance of Cox (early fusion) (fold 5)

^b^ The best performance of Cox (late fusion) (fold 5)

^c^ The best performance of intermediate fusion (fold 4)

^d^ The best performance of late fusion (fold 5)

^e^ The best performance of block super diagonal tensor fusion (fold 4)

^f^ The performance of multimodal factorized higher-order pooling is the same

^g^ Training

^h^ Validation

^i^ Pre-contrast CT

^j^ Post-contrast CT

^k^ Testing

^l^ Approach 1

^m^ Approach 2

^n^ Approach 3

^o^ The performance of the most predictive model (combine the *t*-test selection method and an SVM as a classifier), training set

^p^ The performance of the most predictive model (combine the *t*-test selection method and an SVM as a classifier), validation set

^q^ Multi-view

^r^ Multi-view score

^s^ The best performance of multi-view (SVM-based)

^t^ The best performance of multi-view score (SVM-based)

For the radiomic models in eight articles, the area under the receiver operating characteristic curve (AUC) was used to evaluate the performance in four studies [26, 30, 33, 35]. And the *C*-index was used to evaluate the performance in four studies [27, 28, 31, 35]. One of the articles did not show the performance [34].

For the genomic models in five articles, the AUC was used to evaluate the performance in three studies [26, 30, 35]. The *C*-index was used to evaluate the performance in three studies [28, 31, 35].

The AUC was used for combination models to evaluate the performance in four studies [26, 30, 33, 35], ranging from 0.72 to 0.99. Notably, all AUC values from combined models were higher than their respective independent models. The *C*-index was used to evaluate the performance in seven studies [27–29, 31, 32, 34, 35].

### Risk of bias assessment

As shown in Supplementary Table 1, the mean RQS of the study is 12.2 (range 2–23). All the papers contain discriminant statistics, but only 20% (2/10) [29, 34] of the studies included calibration statistics. In 90% of the manuscripts [26, 27, 29–35], feature reduction was carried out to explain the possibility of overfitting. Importantly, all studies have the verification, 80% of which use internal testing [26, 28–32, 34, 35]. The ROB results and the assessment of the applicability of these studies are shown in Supplementary Table 2. Overall, all included articles were regarded as having a high ROB.

## Discussion

In this review, we summarized and compared the methods of establishing prognostic models by combining radiomics and genomics. The results showed that the combination model can potentially improve prognosis prediction performance.

From the overall results, all models that combine radiomics and genomics have good predictive performance. Among them, the performance of three articles was better than that of radiomics and genomics [26, 30, 31], two articles were better than radiomics (no genomic model) [27, 33], and three articles were not compared because there was no performance of single model [29, 32, 34]. In particular, the combination model for predicting the adjuvant chemotherapy response in one of the articles was superior to the individual model, while the combination model for predicting OS was not necessarily [35]. Due to variations in fusion methods, the performance of the combination models in two article fluctuated, sometimes surpassing, and sometimes falling short of that of a single model [28, 35]. Although the combined model enhanced prognostic prediction, additional research is needed to explore this finding further.

In the articles we included, the least absolute shrinkage and selection operator (LASSO) method [36] was the most common method for radiomic feature selection (6/10, 60%) [29–32, 34, 35], and two are also used to select genomic features [30, 35]. In addition, Bi-clustering, consensus clustering, the ReliefF algorithm, and the statistical method accounting for relevancy and redundancy were also mentioned [27, 32, 33]. One of the articles specifically compared the effects of three feature selection methods on the performance of the model [32]. It was discovered that the Lasso method indicates the best classification effect [32]. However, the number of radiogenomic features formed by the combination was only explicitly mentioned in three articles, ranging from 3 to 20 [26, 30, 33]. Moreover, multivariate Cox proportional hazard analysis is mostly used to determine predictive factors (5/10, 50%) [29–32, 34], which is computationally efficient and can find the relationship between features [37, 38]. So, it seems that using the Lasso method and multivariate Cox proportional hazard analysis may be a common approach. However, the most accurate model has an AUC of 0.95 on the training set and 0.99 on the verification set, obtained by combining the *t*-test selection method and support vector machine (SVM) as a classifier [33]. Moreover, some studies have analyzed the advantages and disadvantages of various selection methods under the traditional mode, which may provide reference to some extent [39, 40]. Therefore, future research should continue exploring the scope of applying different feature selection methods to establish a more accurate model.

The model established by deep learning methods [41] was not common [28, 35]. Though the SVM-based model showed superior performance [35], some other combination methods cannot fully improve the performance [28, 35]. This may be caused by overfitting caused by small data sets [28]. Because deep learning methods can improve radiomics but are still developing [42–44], the application of deep learning in the combination model must be further explored.

Some articles combined radiomics, genomics, and clinical information to get more accurate prognostic results [27, 30–32, 34], which involves multimodal fusion [45]. All articles with clinical data showed that a combined model with three factors had high levels of prediction accuracy. Furthermore, clinical data can be modeled with risk scores after calculating radiomics and genomics [31], which differs from the direct combination method but also shows good performance. Therefore, the differences between various computational methods and prognostic models can be considered in future research.

In addition, few studies [27, 29, 34] have shown the relationship between radiomics and genomics, and the biological basis of radiomics can be explored by gene enrichment analysis. The results suggest that the biological basis of the radiomics model may be due to changes in immune and metabolic pathways, which could provide more potential for prognosis prediction of lung cancer [46, 47].

This review has some limitations. First, a common limitation across these studies is the retrospective design, which may introduce selection biases and limit the universality of the results. Second, most articles contain a small sample size [26, 28–34], and insufficient sample size may affect the robustness and bias of the model [15, 31]. The need for standardized methods and data accessibility are also challenges for radiogenomics. Cloud-based solutions may help manage large datasets, which requires the creation of a trusted research environment within the necessary governance [48]. Future research should focus on multicenter, prospective studies that can validate these findings in a more heterogeneous patient cohort. Finally, studies also need to explore the practical integration of these predictive models into clinical workflows, ensuring they are accessible and interpretable to clinicians.

## Conclusion

This review found that in most studies, the combination model of radiomics and genomics performed better than the prognostic model formed by radiomics or genomics alone. The combination of radiomics and genomics may be helpful in predicting the prognosis of lung cancer and provide new support for patients’ treatment strategies. Prospective and detailed studies should be conducted to determine and promote the combined approach’s advantages.

## Supplementary information

ELECTRONIC SUPPLEMENTARY MATERIAL

## Abbreviations

AUC: Area under the receiver operating characteristic curve
*C*-index: Concordance index
DFS: Disease-free survival
LASSO: Least absolute shrinkage and selection operator
ORR: Objective response rate
OS: Overall survival
PFS: Progression-free survival
PROBAST: Predictive model bias risk assessment tool
RNA: Ribonucleic acid
ROI: Region of interest
RQS: Radiomics quality score
SVM: Support vector machine
