# Supplementary material for: The prognostic value of radiogenomics using CT in patients with lung cancer: a systematic review
Source: Insights Imaging. 2024 Oct 28;15:259. doi: 10.1186/s13244-024-01831-4 (PMC11519241; doi:10.1186/s13244-024-01831-4)
Supplement: Supplementary file 1 — ELECTRONIC SUPPLEMENTARY MATERIAL [file 13244_2024_1831_MOESM1_ESM.docx]

**Supplementary Table 1** The radiomics quality score (RQS) of the included studies

| Study ID | Image  protocol  quality  (0–2) | Multiple segmentations  (0 or 1) | Phantom study  on all scanners  (0 or 1) | Imaging at multiple  time points  (0 or 1) | Feature reduction  or adjustment  (−3 or 3) | Multivariable analysis with non radiomics features  (0 or 1) | Biological  correlates  (0 or 1) | Cut-off  analyses  (0 or 1) | Discrimination  statistics  (0–2) |
| --- | --- | --- | --- | --- | --- | --- | --- | --- | --- |
| Nastaran Emaminejad, 2016 [26] | 0 | 0 | 0 | 0 | 3 | 1 | 1 | 1 | 1 |
| Patrick Grossmann, 2017 [27] | 0 | 1 | 0 | 0 | 3 | 1 | 1 | 0 | 2 |
| Vaishnavi Subramanian, 2020 [28] | 0 | 1 | 0 | 0 | -3 | 1 | 0 | 0 | 1 |
| Liyuan Fan, 2020 [29] | 1 | 1 | 0 | 0 | 3 | 1 | 1 | 1 | 1 |
| Ki Hwan Kim, 2020 [30] | 1 | 1 | 0 | 0 | 3 | 1 | 0 | 0 | 1 |
| Wei Chen, 2022 [31] | 0 | 1 | 0 | 0 | 3 | 1 | 0 | 1 | 2 |
| Apurva Singh, 2022 [32] | 0 | 0 | 0 | 0 | 3 | 1 | 0 | 0 | 2 |
| Amine Bouhamama, 2023 [33] | 0 | 0 | 0 | 0 | 3 | 1 | 0 | 0 | 1 |
| Qi‐Kun Guo, 2023 [34] | 1 | 1 | 0 | 0 | 3 | 1 | 1 | 1 | 2 |
| Eleftherios Trivizakis, 2023 [35] | 0 | 1 | 0 | 0 | 3 | 1 | 0 | 0 | 2 |
|  |  |  |  |  |  |  |  |  |  |
| Study ID | Calibration  Statistics  (0–2) | Comparison to the gold standard  (0 or 2) | Potential clinical  utility  (0 or 2) | Cost-effectiveness analysis  (0 or 1) | Open science and data  (0–4) | Total points 1  (-7 to 24) (%) | Prospective  study  (0 or 7) | Validation  (−5 to 5) | Total points 2  (−12 to 36) (%) |
| Nastaran Emaminejad, 2016 [26] | 0 | 0 | 0 | 0 | 0 | 7 | 0 | 2 | 9 |
| Patrick Grossmann, 2017 [27] | 0 | 2 | 0 | 0 | 1 | 11 | 0 | 3 | 14 |
| Vaishnavi Subramanian, 2020 [28] | 0 | 0 | 0 | 0 | 2 | 2 | 0 | 2 | 4 |
| Liyuan Fan, 2020 [29] | 1 | 2 | 2 | 0 | 0 | 14 | 7 | 2 | 23 |
| Ki Hwan Kim, 2020 [30] | 0 | 2 | 0 | 0 | 0 | 9 | 0 | 2 | 11 |
| Wei Chen, 2022 [31] | 0 | 2 | 0 | 0 | 1 | 11 | 0 | 2 | 13 |
| Apurva Singh, 2022 [32] | 0 | 2 | 0 | 0 | 0 | 8 | 0 | 2 | 10 |
| Amine Bouhamama, 2023 [33] | 0 | 0 | 0 | 0 | 0 | 5 | 0 | 3 | 8 |
| Qi‐Kun Guo, 2023 [34] | 1 | 2 | 2 | 0 | 0 | 15 | 0 | 2 | 17 |
| Eleftherios Trivizakis, 2023 [35] | 0 | 2 | 0 | 0 | 2 | 11 | 0 | 2 | 13 |

Total point 1 indicates the Radiomics quality scores that excluded the items about prospective study and validation; Total point 2 indicates the scores calculated for all 16 items.

**Supplementary Table 2** Radiomics quality scores for the included studies

| Study ID | Risk |  |  |  |  | Applicability |  |  |  | Overall |  |
| --- | --- | --- | --- | --- | --- | --- | --- | --- | --- | --- | --- |
|  | Participants | Predictors | Outcome | Analysis |  | Participants | Predictors | Outcome |  | ROB | Applicability |
| Nastaran Emaminejad, 2016 [26] | - | ? | ? | - |  | + | + | ? |  | - | ? |
| Patrick Grossmann, 2017 [27] | - | ? | ? | - |  | + | + | ? |  | - | ? |
| Vaishnavi Subramanian, 2020 [28] | - | ? | ? | - |  | + | + | ? |  | - | ? |
| Liyuan Fan, 2020 [29] | + | ? | ? | - |  | + | + | ? |  | - | ? |
| Ki Hwan Kim, 2020 [30] | - | ? | + | - |  | + | + | + |  | - | + |
| Wei Chen, 2022 [31] | - | ? | ? | - |  | + | + | ? |  | - | ? |
| Apurva Singh, 2022 [32] | - | ? | ? | - |  | + | + | ? |  | - | ? |
| Amine Bouhamama, 2023 [33] | - | + | ? | - |  | + | + | ? |  | - | ? |
| Qi‐Kun Guo, 2023 [34] | - | ? | + | - |  | + | + | + |  | - | + |
| Eleftherios Trivizakis, 2023 [35] | - | ? | ? | - |  | + | + | ? |  | - | ? |

+ indicates low ROB/low concern regarding applicability; − indicates high ROB/high concern regarding applicability; ? indicates unclear ROB/unclear concern regarding the applicability

**Supplementary Table 3** The details of the acquisition parameters of the images and features

| **First author** | **Contrast CT** | **Collection time** | **CT tube voltage** | **CT tube current** | **Segmentation software** | **Feature extraction algorithm/software** | **Type of radiomic features** |
| --- | --- | --- | --- | --- | --- | --- | --- |
| Nastaran Emaminejad [26] | / | Before surgery | 120-140KV | 140-340mAs | / | / | Morphological and CT number distribution,  Texture feature |
| Patrick Grossmann [27] | YES or NO^a^ | Before surgery | / | / | / | / | First-order statistics,  Shape and size,  GLCM, RLGL, GLSZM,  Wavelet,  LOG feature |
| Vaishnavi Subramanian [28] | / | Before surgery | 80–140 kVp | 124–699 mA | DICOM Toolkit dcmtk | PyRadiomic;  Imagenet-pretrained DenseNet | Texture and intensity patterns feature^b^ |
| Liyuan Fan [29] | YES | pretreatment | 120 kV | 190-600 mA | / | LIFEx software | Histogram,  Shape,  GLCM matrix, GLZLM matrix, GLRLM matrix, NGLDM matrix, |
| Ki Hwan Kim [30] | YES^c^ | Before surgery | 80-140 kVp | 150-200 mA | MRIcro^d^ | Pyradiomics;  MATLAB^e^ code | Histogram-based,  Shape-based,  GLCM-based,  ISZM-based feature |
| Wei Chen [31] | / | Before surgery | 80-140 kV | 124-699mA | / | Pyradiomics | First-order statistics,  Shape,  GLCM, GLDM, GLRLM, GLSZM, NGTDM,  Wavelet feature |
| Apurva Singh [32] | / | Before surgery | / | / | ITK- SNAP^f^ | Cancer Imaging Phenomics Toolkit | Intensity or first-order statistics, Histogram,  Volumetric,  Morphologic,  GLRLM,  NGTDM,  GLSZM,  Local binary pattern feature |
| Amine Bouhamama [33] | / | within one month before treatment | 100-130Kv | 350–700 mAs | ITK-SNAP^g^ | / | Size,  Shape,  Texture feature |
| Qi‐Kun Guo [34] | Yes | within two weeks (median value of 6 days) | 120 kVp | 500 mA (Max) | ITK-SNAP^h^ | PyRadiomics^i^ | Shape,  First-order intensity statistics,  Texture,  LOG,  Wavelet feature |
| Eleftherios Trivizakis  [35] | / | Before surgery | 80–140 kVp | 124–699 mA | / | PyRadiomics | First-order  Shape  GLCM,GLDM,GLRLM,GLSZM,NGTDM |

GLCM: Gray level cooccurrence matrix; GLDM: gray level dependence matrix; GLRLM: gray level run length matrix; GLSZM: gray level size zone matrix; ISZM: intensity size zone matrix; LOG: Laplacian of Gaussian; NGTDM: Neighboring Gray Tone Difference Matrix; RLGL: run-length gray-level matrix; a: with either contrast CT or non-contrast CT; b: Its immediate surrounding tissues of the lung; c: pre-contrast CT and post-contrast CT; d: version 1.40, Chris Rorden, University of Nottingham, UK; e: MATLAB 2017b, Mathworks Inc, MA, USA; f: v 3.6.0; g: [www.itksnap.org](http://www.itksnap.org); h: version 3.8.0, https://www.itksnap.org; i: version 3.8.3, <https://www.python.org>

**Supplementary Table 4** The number of the gene features for combination model

| **First author** | **Number of genes** | **Number of genes after selection** |
| --- | --- | --- |
| Nastaran Emaminejad [26] | 2 genomic biomarkers (ERCC1, RRM1) | 2 genomic biomarkers (ERCC1,RRM1) |
| Patrick Grossmann [27] | / | / |
| Vaishnavi Subramanian [28] | 5268 genes (RNA-sequencing) | Top 500 |
| Liyuan Fan [29] | / | 2^a^/2^b^/1^c^ |
| Ki Hwan Kim [30] | / | 6^d^/7^e^ |
| Wei Chen [31] | / | 10 |
| Apurva Singh [32] | 36 | 11^f^/7^g^/1^h^ |
| Amine Bouhamama [33] | 2559 | 16/19 |
| Qi‐Kun Guo [34] | 1 | 0 |
| Eleftherios Trivizakis  [35] | 5268 | 3^i^/7^j^ |

a: for ORR; b: for OS; c: for PFS; d: pre-contrast CT scan; e: post-contrast CT scan; f: Approach 1; g: Approach 2; h: Approach 3; i: for therapy response; j: for OS;
